# Evaluating the Carcinogenic and Non-Carcinogenic Health Risks of Heavy Metals Contamination in Drinking Water, Vegetables, and Soil from Gilgit-Baltistan, Pakistan

**DOI:** 10.3390/toxics13010005

**Published:** 2024-12-25

**Authors:** Nafeesa Khatoon, Sartaj Ali, Azhar Hussain, Jia Huang, Zengli Yu, Hongyan Liu

**Affiliations:** 1 Department of Medical Genetics, Henan Provincial People’s Hospital, Zhengzhou 450003, China; nafeesaali.ft1354@gmail.com (N.K.); xxhj0712@126.com (J.H.); 2 College of Public Health, Zhengzhou University, Zhengzhou 450001, China; 3 Department of Agriculture & Food Technology, Karakorum International University Gilgit, Gilgit 15100, Pakistan; sartaj@kiu.edu.pk (S.A.); azhar.hussain@kiu.edu.pk (A.H.)

**Keywords:** environmental pollutants, toxic metal, health risk, cancer, exposure pathways, non-carcinogenic risk, mountain region

## Abstract

Environmental pollutants significantly impact health and quality of life. High levels of harmful metals in drinking water, vegetables, and soil can accumulate in the body, leading to serious health issues. In Gilgit-Baltistan, Pakistan, the prevalence of cancer is notably high, and heavy metals are considered among the possible risk factors. In this study, the distribution of heavy metals, e.g., Cd, Mn, Cu, Cr, and Ni, in the drinking water, vegetables, and soil in the Gilgit and Skardu Districts was assessed. A geo-accumulation index was combined with a deterministic technique to examine potential carcinogenic and non-carcinogenic impacts on human health. Cr and Ni levels in drinking water exceeded the permissible limits from both districts. Drinking water had a HQ > 1 for Cd, Cr, Ni, and Mn, posing significant non-carcinogenic health effects. The lifetime cancer risk (LCR) by drinking water for Cd, Cr, and Ni in Gilgit (0.0087, 0.55, and 0.0048) and Skardu (0.071, 0.21, and 0.014) indicated a considerable cancer risk (>1 × 10^−4^) for adults. The cancer risk for vegetable intake was observed within the safe limit, while LCR_ingetsion > LCR_dermal and LCR_inhalation for the soil samples in both regions. These findings highlight the need for regular monitoring, enhanced waste management, and advanced purification methods to reduce cancer risk, lower heavy metal contamination, and safeguard public health in the region.

## 1. Introduction

The accumulation of potentially toxic metals such as cadmium (Cd), nickel (Ni), chromium (Cr), copper (Cu), and manganese (Mn) in water, food, and soil has become a major global public health concern [1,2]. These metals are naturally present, and anthropogenic activities, i.e., agricultural practices, inappropriate waste management, and industrial operations, lead to elevated levels of these metals in the environment [3]. The consumption of certain toxic metals, such as copper (Cu), zinc (Zn), cobalt (Co), iron (Fe), and manganese (Mn), are vital for human health [4]. These metals are necessary for biological signaling pathways and diverse metabolic processes. Nevertheless, essential metals can also be toxic and carcinogenic if they are consumed in excess amounts [5]. However, even at low levels, other heavy metals such as cadmium (Cd), nickel (Ni), lead (Pb), mercury (Hg), and chromium (Cr) can have harmful effects. Possible adverse effects include cancer, cardiovascular disease, hypertension, and severe intellectual disabilities [6,7]. A heavy metal’s primary concern is its distinct properties, such as toxicity, persistence, and potential for bioaccumulation [8]. Toxic heavy metals are recognized as posing serious health concerns to humans because of their propensity to accumulate in living things and to persist in the environment [9].

Cd, Cr, and Ni are classified as Group 1 carcinogens by the International Agency for Research on Cancer (IARC 1999). It has been demonstrated that cadmium exposure significantly contributes to lung, breast, prostate, kidney, and pancreatic cancers. As a result of altering epigenetic pathways, disrupting cell signaling mechanisms, causing oxidative stress, and degrading DNA, Cd can stimulate carcinogenesis [10]. In addition to inhibiting DNA repair, Cd contributes to the progression and growth of cancer [11]. Among humans, Cr can cause nasal, stomach, and lung cancers by altering tumor suppressor genes. It causes chromosomal scratches and induces DNA damage by producing reactive oxygen species (ROS) and contributing to mutagenesis [12]. Ni promotes cancer in the nose, lungs, and throat by producing ROS, compressing tumor suppressor genes, modifying DNA methylation, and altering epigenetic factors. As a result of these mechanisms, DNA and cells are incapable of repairing themselves, leading to cancer development [13]. Mn is not directly linked with cancer, but excessive accumulation of Mn extended times can induce oxidative stress and neurotoxicity, which can encourage cancer progression in the presence of other toxic metals like Cd, Ni, and Cr [14].

In the past 70 years, there have been 9.6 million cancer-related deaths worldwide, with 18.1 million new cases of the disease being diagnosed [15,16]. According to the statistics, 91 out of 172 countries have cancer as the leading cause of death, which is a frightening distinction [17]. In 2040, there will be 29.5 million cancer diagnoses along with 16.3 million deaths from the disease [18]. Global cancer rates are rising as a result of new issues with rapid urbanization, aging populations, inactivity, unhealthy lifestyles, and environmental degradation [19,20].

A significant relationship was found between thyroid cancer and heavy metal exposure. A noticeable level of toxic metals was observed in the urine samples of residents in Sicily, Mount Edna [4]. A recent study also assessed heavy metals concerning breast cancer from five major continents all over the world and revealed that all the biological specimens had higher levels of toxic metals [21]. The alarming relationship between the disclosure of these toxic metals and the elevated prevalence of various cancers within affected populations has been underlined by recent epidemiological studies [22,23].

The United State Environmental Protection Agency (USEPA) issued health indices, which are important tools for quantifying and explaining potential adverse effects of heavy metal exposure on individuals and populations [24]. A comprehensive risk profile can be obtained by integrating exposure levels; toxicity reference values; and individual sensitivities such as the total hazard quotient (THQ), hazard index (HI), and lifetime cancer risk (CR) [25]. The HI facilitates the identification of high-risk exposure situations through a standardized assessment process, allowing public health authorities and policymakers to implement targeted interventions [26]. In addition, these tools and techniques increase the accuracy of health risk assessments and improve environmental health management decisions through assessing different heavy metals and exposure pathways [27]. Its application is critical in developing regions, where industrial activities and environmental degradation often lead to severe heavy metal pollution, putting human health at risk [28].

People who live in places with a high concentration of heavy metal pollution are especially at high risk. The probability of exposure to contaminated water, food, and soil rises significantly in areas with an overflowing density of industrial activities or low environmental laws [29] as reported by Yakamercan et al. (2021). These hazardous metals can enter the body via impure water, food, or direct contact with contaminated soil. Once inside, they can build up in important organs and tissues [30].

Gilgit-Baltistan (GB) lies in the foothills of the mighty Karakoram and Himalaya Mountains and occupies a total area of 72,496 square kilometers. The area is landlocked, roughed and mountainous, and is home to around 2 million people [31]. The nature of the area makes it inaccessible to modern healthcare and educational resources, and the people rely on local mining, agricultural, and tourism sectors for their socioeconomic survival. Every day, the populace is plagued by potentially fatal illnesses like cancer, heart arrest, hepatitis, and renal failure. The number of cancer patients increases, and it is becoming more widespread in the area. The frequency of these illnesses may be related to various causes, including toxic metals, inadequate sanitation and hygiene, food poisoning, and environmental pollution.

The present study therefore aims to examine the concentrations of Mn, Cr, Ni, Cd, and Cu in drinking water, vegetables, and soil from two districts: Skardu and Gilgit, Northern Areas, Pakistan. The core objective is to assess and measure the elevated levels of toxic metals that pose serious threats to public health in the region. It further intends to investigate the possible links of heavy metals to the higher cancer prevalence in the area by applying a combined deterministic approach and chemometric analysis, as well as to explore carcinogenic and non-carcinogenic health risks. As a result of this study, we will gain a greater understanding of the environmental variables contributing to cancer incidence through the contamination of toxic metals. These findings will provide guidance in policymaking and the formulation of public health programs aimed at reducing the exposure levels and minimizing the health hazards associated with heavy metals pollution.

## 2. Materials and Methods

### 2.1. Study Area

GB is a sparsely populated high-mountain region, located in the north of Pakistan. It borders internationally with China in the north, India in the east and southeast, and Afghanistan in the northwest. These districts are located between latitudes 30° N and 37° N and longitudes 72° E and 77° E [32]. Superlatives are often used to characterize its natural surroundings: It has the longest glaciers outside of the Arctic area, home to the second-highest mountain (K_2_) in the world, and has five eight-thousanders [33]. Geographically, it is covered with high mountain ranges: the Hindukush, Karakoram, and Himalayas. GB society is diverse in terms of language, religion, and cultural backgrounds. The region’s physiography varies with elevation, precipitation, amount of snowfall, and sunlight [34]. Ninety-five percent of the population relies on agriculture, the mining sector, and handed-down trade for their livelihood. On tiny farmlands, they raise crops and vegetables irrigated with water from glacier streams, rivers, and springs.

### 2.2. Sampling Plan

**Figure 1:**
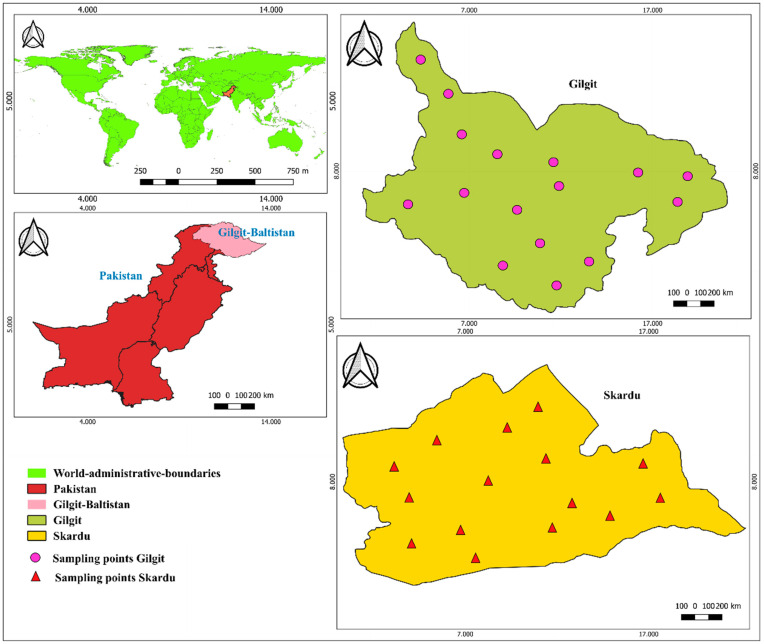
Study area map of the sampling sites.

Specific sites were selected based on the high prevalence of cancer patients in the Gilgit-Baltistan region. First, we collected information about cancer prevalence from the local health authority (Directorate of Health Gilgit-Baltistan), Chief Ministers Health Endowment Fund under the Social Protection Unit. Using this primary information, we selected specific districts (Gilgit and Skardu) based on registered cases of cancer patients from various cancer hospitals in Pakistan. Several factors were taken into account when selecting sampling sites, including population density, agricultural activities, and the use of natural water for drinking and irrigation. Areas with intensive farming practices and higher populations were prioritized due to their substantial contribution to elevated levels of heavy metals as a result of anthropogenic practices. The geogenic contribution to metal contamination has also been considered in sites adjacent to natural mineral deposits, which are particular characteristics of the Gilgit-Baltistan region. Samples (*n* = 175) were collected by a simple random sampling method from purposefully dispersed cancer risk sites in the districts to determine the risk factors of cancer (Figure 1). Four types of leafy vegetables were selected for the current study: spinach, water cabbage, lettuce, and coriander.

The same relative mass of vegetables was collected in triplicate from four central sites in each district. Samples were collected during July and August 2023, when vegetable growth and harvest were at their peak. The edible leafy parts of the vegetables were separated and packed in polyethylene bags.

A total of 60 vegetable samples were collected for toxic metals analysis. Samples of soil (*n* = 55) were collected using an auger from the same field sites where vegetables were selected. Generally, the depth of sampling ranged from 0 to 20 cm, which is the depth at which roots propagate. Soil samples were placed inside polyethylene bags to prevent any possible cross-contamination. Drinking water samples of 500 mL were collected in polyethylene bottles in triplicate from four valleys in each district (*n* = 60). Pre-washing with detergent was followed by at least three rinses with deionized water (DW). During the process of filling the bottles, tap water was left flowing for two minutes prior to filling. Whatman No. 42 filter paper was used to filter the samples. Then, nitric acid (1.5 mL, 65%) was added to maintain the pH of the water, and it was stored at 4 °C for further analysis.

Samples of drinking water, vegetables, and soil were carefully collected, transported, and stored in the Processing Laboratory to avoid possible cross-contamination. The chosen heavy metals were subjected to analysis using slightly modified techniques from previously published reports [35,36].

### 2.3. Sample Preparation

Sample preparation and digestion procedures were carried out following the protocols outlined in the Standard Methods for Examination of Water and Wastewater [37,38]. Initially, samples were prepared in the Processing Laboratory of the Department of Agriculture and Food Technology in Gilgit, Pakistan. In the first step, soil samples were air-dried, ground, and filtered via a stainless steel sieve (22 mm) and then stored at room temperature in a polyethylene bag until they were transferred to the Centralized Resource Laboratory (CRL), University of Peshawar, Pakistan.

A total of two kilograms of vegetables were collected in triplicate from target points and packaged in polyethylene bags. Each vegetable was cleaned with tap water first, followed by three rounds of washing with deionized water to eliminate other impurities. The vegetables were cut into tiny pieces with stainless steel knives and allowed to air dry for 2 h. After being air-dried, samples were placed on a silica plate and dried in an oven at 60 °C. The dried samples were pounded in an electric grinder (Panasonic MX-GM 1011, Kadoma, Osaka, Japan) to the point that it could no longer pass through a 2 mm filter, then placed in desiccators for further use. The collected samples were properly mixed to ensure that each site received a representative sample.

### 2.4. Assessment of Heavy Metal

#### 2.4.1. Measurement of Heavy Metals in Vegetables and Water

The digestion of vegetables and drinking water samples followed the acid digestion procedures as per method 3030E and method 3111B detailed in the Standard Methods for the Examination of Water and Waste Waters [38]. A vegetable sample of one gram was appropriately powdered and incinerated in an electric furnace at a temperature of 250 °C. Then, the incinerated sample was dissolved in HCl to prepare a solution for the identification of toxic metals by an Atomic Absorption Spectrometer (AAS-700, PerkinAlmer, Waltham, MA, USA) [39]. The same analytical instrument was utilized to determine the particular toxic metals from the prepared drinking water sample. AAS (AAS-700) was calibrated with relevant grade standard solutions before sample analysis. The sample solution was diluted once more if the distribution was higher than the calibration limit [40].

#### 2.4.2. Measurement of Heavy Metals in the Soil

The levels of toxic metals in the prepared soil samples were determined by the extraction of hydrogen chloride, nitric acid, hydrogen fluoride, and perchloric acid (HCl-HNO_3_-HF-HCLO_4_), respectively. Three milliliters of HCl (37%), one milliliter of HNO_3_ (65%), six milliliters of HF (65%), and half of one milliliter of HCLO_4_ (65%) were taken with approximately 100 mg of soil sample for digestion. This digestion process follows two stages based on the period to reach 200 °C. Stage 1 takes 10 min, while stage 2 consumes 15 min at the same temperature [36]. The digested sample was evaporated to make them dry after cooling. Then, 10 mL deionized water and 1 mL of 65% HNO_3_ were added. The sample was kept on a heater until an outgrowth of HCLO_4_ steam. After cooling down, 5 mL HF was poured into the crucible, and the contents were dried in a sand bath at 200–225 °C. Water (2 mL) and a few drops of HCLO_4_ were poured into the crucible when the temperature of the crucible was down. The crucible was once more exposed to a sand bath to dry the sample. Subsequently, deionized water (5 mL) and normal HCl (5 mL) were poured into a crucible after some other cooling and then put on the heater for further boiling. Finally, the sample was diluted with water when the residual soil was completely digested in HCl in a 50 mL container. AAS-700 was used for the determination of selected heavy metals from the sample.

### 2.5. Quality Assurance and Control

Quality assurance and quality control are needed in analytical procedures for accuracy and precision. The acid solutions, reagents, standard stock solutions, and multi-metal solutions (Wako Pure Chemical Industries, Osaka, Japan) were purchased from Hajvery Scientific Store in Lahore Pakistan, which was supplied by Merck (Darmstadt, Germany). Calibration graphs were determined using standard metal ion solutions (Agilent Technologies, Stevens Creek Blvd Santa Clara, CA, USA) and a blank solution (0.1 N HNO_3_). The correlation coefficients of the calibration lines for each metal were found to be greater than 0.97. A standard procedure was employed for determining the limits of detection (LoD) and limits of quantitation (LoQ) for each toxic metal ion [41]. A LoD of 3.3 Sa/b was used to express accuracy measures, and a LoQ of 10 Sa/b was used to express precision. Where Sa represents the standard deviation of absorbance, and b shows a slope of the calibration curve [42]. Each sample was assessed three times to determine the consistency of the analysis. In the current study, the recovery rates ranged from 92% to 106%, which is within the satisfactory range of 80% to 120% (Table 1). To ensure reliability and accuracy, the certified reference materials (CRMs) used for vegetable, water, and soil were NIST SRM1640a, NIST SRM1570a, and NIST SRM2709a (San Joaquin Soil).

### 2.6. Health Risk Assessment by a Deterministic Approach

#### 2.6.1. Non-Carcinogenic Risk Assessment

##### Chronic Daily Intake (CDI)

The chronic daily intake (mg/kg/day) was calculated by the formula reported in the USEPA framework for metals risk assessment [35,43] as presented in Equation (1).

(1) $$CDI=\frac{C\times CF\times F_{ir}\times EF\times ED}{BW\times AT}{\times 10}^{-3}$$

where C is the level of heavy metals in mg/kg of the body weight, CF is the conversion factor of vegetables into dry form (0.08) [44], the average food consumption (300–350 g/person) is denoted by F_ir_ [45], EF (365 day/year) is the average exposure frequency, ED is exposure length 70 years, BW is the mean body weight (70 kg) of the individual, and AT 365 × 70 is the mean period of disclosure. The chronic daily intake of heavy metals through the soil via three major pathways was calculated by using the expressions Equations (2)–(4); the required parameters for calculation of the CDI in soil are shown in Table S1.

(2) $${CDI}_{ing}=\frac{C_{soil}\times IRs\times EF\times ED\times CF}{BW\times AT}$$

(3) $${CDI}_{derm}=\frac{C_{soil}\times SA\times SAF\times ABS\times EF\times ED}{BW\times AT}$$

(4) $${CDI}_{inh}=\frac{C_{soil}\times {IR}_{inh}\times EF\times ED}{BW\times PEF\times AT}$$

##### Total Hazard Quotient

The total hazard quotient is the ratio of chronic daily intake to oral reference dose (RfD) for individual metals in mg/day/kg body weight, presented in Equation (5) [46]:

(5) $$THQ=\frac{CDI}{RfD}$$

There might be a potential adverse health risk if the value of THQ is equal or more than one. THQ less than one indicates that there are no significant adverse health effects of heavy metals in individuals or consumers.

##### Hazard Index

The hazard index (HI) is the numerical value applied in a risk assessment to evaluate the potential non-carcinogenic health outcomes of exposure to hazardous metals. It is used to assess whether the combined exposure to multiple contaminants exceeds the safe levels. It was calculated by summing the hazard quotients (HQs), as presented in Equation (6), for each heavy metal [36,47] accordingly.

(6) $$HI={HQ}_{Mn}+{HQ}_{Cr}+{HQ}_{Cu}+{HQ}_{Ni}+{HQ}_{Cd}$$

If HI < 1 indicates that the combined exposure to the heavy metals is unlikely to pose a considerable risk of non-carcinogenic health outcomes, HI ≥ 1 suggests that there may be potential for detrimental health effects, and a more comprehensive assessment may be required.

The evaluation of the hazard quotient posed by soil via different pathways was calculated by using Equations (7)–(9), as shown below [37]:

(7) $${HQ}_{ing}=\frac{{CDI}_{ing}}{RfD}$$

(8) $${HQ}_{derm}=\frac{{CDI}_{derm}}{RfD}$$

(9) $${HQ}_{inh}=\frac{{CDI}_{inh}}{RfD}$$

### 2.7. Carcinogenic Risk Assessment

Excess lifetime cancer risk (LCR) states the probability that an individual will develop cancer throughout their lifetime due to the disclosure of carcinogenic heavy metals, as presented in Equation (10). This measure helps in understanding the long-term impact of exposure to environmental pollutants such as heavy metals on human health [48,49,50].

(10) LCR=CDI×CSF

CSF is an oral carcinogenic slope factor [51]. A LCR of 1 × 10^−6^ (1 in 1,000,000); is generally considered a negligible risk, while an excess lifetime cancer risk of 1 × 10^−5^ (1 in 100,000) is the limit where the regulatory authorities may consider action. A lifetime cancer risk of 1 in 10^−4^ (1 in 10,000) indicates a higher level of risk that may warrant more regulatory action or remediation efforts.

The lifetime cancer risk of particular heavy metals was assessed by utilizing the mathematical Expressions (11)–(13) [37]:

(11) $${LCR}_{ing}={CDI}_{ing}\times CSF$$

(12) $${LCR}_{derm}={CDI}_{derm}\times CSF$$

(13) $${LCR}_{inh}={CDI}_{inh}\times CSF$$

### 2.8. Geo-Accumulation Index

The Geo-accumulation index (I_geo_) was evaluated to determine the soil pollution and pattern of contamination by using the mathematical Expression (14) [35,52,53]:

(14) $$I_{\mathrm{geo}}={log}_{2}(\frac{C_{n}}{1.5B_{n}})$$

where C_n_ is the level of heavy metal in the soil, B_n_ is the geochemical background value for non-contaminated soil of the study area, and the constant value (1.5) allows for the reduction in the variation in background concentration because of lithogenic consequences. The I_geo_ can be categorized into the following limits to distinguish the quality of the soil. I_geo_ ≤ 0 means the soil is safe and uncontaminated, I_geo_ < 1 indicates the soil is slightly contaminated, I_geo_ < 2 represents moderate contamination, I_geo_ < 3 indicates highly moderate contamination, I_geo_ < 4 represents heavily contaminated, and I_geo_ < 5 indicates extremely contaminated.

### 2.9. Statistical Analysis

Statistical analyses were done in R Studio (Version 2024.09.0+ 375) for descriptive and multivariate analyses, and Origin Pro2024 software was used for graphical representation. The Shapiro–Wilk test was applied to the data for normal distribution. Statistically significant differences in heavy metal concentration between samples with respective districts were assessed with two-way ANOVA followed by Tukey’s test, principal component analysis (PCA), and Pearson’s correlation matrix. The level of statistical significance was set at *p* ≤ 0.05. The Quantum Geographic Information System (QGIS) software (Version 3.36.2) created the study area map using the coordinates of selected target sampling points.

## 3. Results and Discussion

### 3.1. Concentration of Cadmium in Drinking Water, Vegetables, and Soil

**Figure 2:**
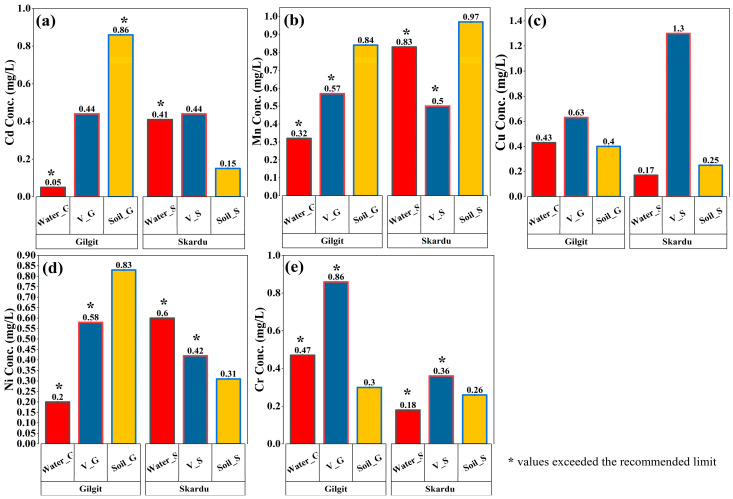
Average distribution of heavy metals in samples across the districts. (**a**) Concentration of Cd in the drinking water, vegetable, and soil samples. The level of Cd exceeded the permissible level set by the FAO/USEPA (0.003 mg/L) and NSDWQ-PAK (0.01 mg/L) in drinking water. (**b**) Concentration of Mn in various samples. The Mn concentrations exceeded the allowable limit set by the WHO and USEPA in both districts in water (0.2 mg/L) and vegetables (0.06 mg/kg). (**c**) The distribution of Cu in various samples was noticeably different at *p* < 0.05. The highest contamination was recorded for the vegetable sample in Skardu, followed by vegetables in Gilgit. The current results are within the allowable limit for Cu set by the WHO and USEPA. (**d**) Distribution of Ni in drinking water, vegetables, and soil samples. The Ni average values are 0.2 mg/L and 0.60 mg/L in water samples, which exceeded the permissible limits in water and vegetables (0.07 mg/L, 0.2 mg/kg). (**e**) Levels of Cr in drinking water, vegetable, and soil samples. Cr observed as 0.47 mg/L and 0.18 mg/L in drinking water samples exceeded the safe limit in both regions, the highest Cr was observed in vegetable samples from the Gilgit region.

There is a significant difference (*p* ≤ 0.05) in the levels of the selected toxic metals in drinking water, vegetables, and soil samples in both districts. Figure 2a demonstrates the mean value of cadmium in drinking water, vegetables, and soil. It was found that the mean concentrations of the Cd were 0.05 mg/L in water, 0.44 mg/kg in vegetables, and 0.86 mg/kg in soil, respectively, from Gilgit District. The results indicated a higher concentration of Cd in the soil sample (0.86 mg/kg) compared to drinking water and vegetables. The same metal was found at 0.41 mg/L, 0.44 mg/kg, and 0.15 mg/kg, respectively, in drinking water, vegetable, and soil samples from Skardu. It is observed that a higher level of Cd was found in vegetable samples than in drinking water and soil. The concentration of Cd in drinking water and soil exceeded the permitted limits set by the United States Environmental Protection Agency (USEPA, 2007) and World Health Organization (WHO). A higher level of Cd in drinking water and soil poses adverse effects to an individual’s health. Conversely, Cd levels in the vegetable and soil samples from Skardu were found within the safe limits. The permissible limits for Cd in water, vegetables, and soil set by the WHO and USEPA are 0.003 mg/L, 0.8 mg/kg, and 0.3 mg/kg, respectively [54]. The value of Cd in the drinking water exceeded the acceptable limit (0.01 mg/L) set by the National Standard Drinking Water Quality-Pak (NSDWQ-PAK) from both study areas [46].

Previous studies have also reported elevated levels of Cd in a water sample that exceeded the WHO-approved threshold [55,56]. It has been further reported that the Cd concentration exceeded the reference level in agricultural soil and commonly consumed vegetables in Ethiopia [35]. Another study conducted by [57] also observed that Cd concentrations were above the tolerable limit in the soil.

Sagagi et al. [58] also found high levels of Cd and Pb in drinking water as the major pollutants affecting the water quality in Southeast Nigeria.

The elevated levels of Cd in the research area may be due to the extensive use of phosphate fertilizers, which contain Cd as an impurity and constitute a source of contamination. These fertilizers may be introduced into rivers and streams in Gilgit-Baltistan by agricultural runoff, increasing the level of Cd in the water. Inadequate waste management practices, such as improper disposal of batteries, electronics, and other products holding Cd, can lead to Cd seeping into the soil and water. In this study area, the rugged topography may make it more difficult for Cd to diffuse through surface runoff and groundwater. The widespread application of fertilizer based on phosphate to increase crop productivity in hilly areas may cause Cd to leak into adjacent water bodies through agricultural runoff. Another important contributing factor in the region is the natural weathering of rocks and minerals that are rich in Cd. The local community lacks a suitable water treatment facility to purify its drinking water, which causes Cd to build up in the water sources.

### 3.2. Concentration of Manganese in Water, Vegetables, and Soil

In the current study, there was a considerable difference (*p* ≤ 0.05) in the level of toxic metal between samples and within districts. It was observed that the mean concentration of Mn was recorded as 0.32 mg/L, 0.57 mg/kg, and 0.84 mg/kg, respectively, in the drinking water, vegetables, and soil from Gilgit District (Figure 2b). The results for the same metal were observed as 0.83 mg/L, 0.50 mg/kg, and 0.97 mg/kg, respectively, in the drinking water, vegetable, and soil samples of Skardu District. It is noted that both soil samples (0.84 and 0.97 mg/kg) showed higher levels of Mn than drinking water and vegetable samples collected from the respective districts (Figure 2b). The obtained results showed that Mn concentrations in water and vegetables exceeded the allowable limits set by the WHO and USEPA in both districts, indicating that the drinking water and vegetables were severally contaminated with Mn. Mn levels allowed by regulatory agencies are 0.2 mg/L for water samples and 0.06 mg/kg for vegetable samples [59].

Ashraf et al. [44] also reported high values of Mn in vegetables ranging from 33.41 to 129.32 mg/kg, surpassing the cutoff point in Kasur District, Pakistan. Another study stated that Mn was the most common pollutant in the soil, irrigation water, and vegetables in Nigeria [58]. Hussain [31] also investigated toxic element pollution, as well as risk to human health, and found that the Mn level in the water was above the recommended limits in Skardu and Gilgit.

Weathering and erosion of manganese-rich rocks could be attributed to the elevated concentrations of Mn in the studied regions. After that, the metal is discharged into soil and water sources for plant absorption. The Mn content in the soil increases as a result of the application of fertilizers and soil additives [60]. Even though Mn plays a vital function in bone formation, antioxidant enzyme activity, metabolism, and limiting exposure to safe are is essential [61]. Overexposure to this substance can pose serious health risks, especially to the nervous system.

### 3.3. Level of Copper in Water, Vegetables, and Soil

It was noticed that the level of Cu was meaningfully different (*p* ≤ 0.05) among various samples and within the district. Figure 2c demonstrates the average concentration of Cu in the drinking water, vegetables, and soil samples, respectively. The mean concentration of Cu was observed as 0.43 mg/L, 0.63, and 0.40 mg/kg, respectively, in the drinking water, vegetable, and soil from Gilgit District. The trend for the same metal from Skardu District was noticed as 0.17 mg/L, 1.3 mg/kg, and 0.25 mg/kg, respectively, in drinking water, vegetables, and soil. The highest level of Cu was found in vegetable samples for both districts, which demonstrated that the vegetable sample was substantially contaminated by Cu. On the other hand, a very low level of 0.17 mg/L was recorded for the water sample, followed by vegetables from Skardu. The current results are within the allowable limit for Cu set by the WHO and USEPA [62], so a tolerable upper-level intake of Cu is 10 mg/day. The results indicated that the drinking water, vegetables, and soil were not significantly contaminated by Cu, which means that they could not pose considerable adverse health outcomes in the community.

Previous studies have also reported similar results of Cu having elevated levels in Cuban soils [63]. A review paper concludes that heavy metal contamination of water, soils, and vegetables poses a serious threat to health by inducing toxicity in the food and environment [64]. The Cu content of the water, vegetables, and soil was found to be within the acceptable range in another investigation on possible health risk factors [36].

### 3.4. Concentration of Nickle in Water, Vegetables, and Soil

A statistically significant difference was observed in nickel distribution between sample types at *p* < 0.05; meanwhile, the interaction between samples and district was also statistically noteworthy. The observed *p*-value (*p* ≤ 0.05) indicates a statistically significant difference in Ni concentrations among Gilgit and Skardu Districts. This suggests that local environmental factors and human activity may contribute to the higher contamination levels observed in Skardu. It was found that the mean concentration of Ni was 0.20 mg/L, 0.58 mg/kg, and 0.83 mg/kg, respectively, in drinking water, vegetables, and soil from Gilgit District (Figure 2d). The trend for the same metal was noticed as 0.60 mg/L, 0.42, and 0.31 mg/kg, respectively, in the drinking water, vegetables, and soil samples from Skardu District. A maximum quantity of Ni was found in the soil sample of Gilgit, and the drinking water sample of Skardu noticeably showed a maximum level of Ni. The results revealed that Ni concentration in the drinking water and vegetables from both districts surpassed the permissible level set by the WHO and USEPA (WHO 2011). The recommended allowable limit for drinking water and vegetables is 0.07 mg/L and 0.2 mg/kg [65]. Furthermore, the NSDWQ-PAK established the guideline level of Ni 0.02 mg/L [66]. According to the International Agency for Research on Cancer (IARC), Ni is considered carcinogenic to humans and categorized as Group 1, leading to cancer of the lungs, nasal cavity, and paranasal sinuses [67].

Many studies have shown that that Ni, Mn, and Cr were above the WHO/FAO safe range in soil, vegetables, and plant crops [68]. Similarly, Wang et al. [69] noticed a higher level of Ni in the groundwater in Guanzhong Basin, China [70]. Another study by Hussain et al. [31] also recorded the highest concentration of Ni in the drinking water, causing carcinogenic health effects [46].

### 3.5. Distribution of Chromium in Drinking Water, Vegetables, and Soil

The observed *p*-value of ≤0.05 indicated a statistically significant difference in Cr concentration between Gilgit and Skardu Districts (Figure 2e). The mean concentration of Cr was observed as 0.47 mg/L, 0.86 mg/kg, and 0.30 mg/kg, respectively, in the drinking water, vegetables, and soil from Gilgit District. The values for Skardu District were recorded as 0.18 mg/L, 0.36 mg/kg, and 0.26 mg/kg, respectively, in the drinking water, vegetables, and soil samples. The results demonstrated that Gilgit District had the highest quantity of Cr for all the samples, whereas the lowest concentrations were found in the Skardu region. While comparing the drinking water, and soils samples, vegetables had the maximum concentration of Cr from both areas. It was observed that drinking water samples were highly contaminated by Cr and significantly exceeded the permissible level (0.05 mg/L) [40]. These observations indicate a serious public health concern for both the regions. The exceeding levels may be due to the local environmental factors such as mining activities and agricultural runoff that have contributed to higher contaminations in Gilgit and Skardu. When compared to previous studies in other mountainous regions, the levels observed in Gilgit and Skardu were among the highest reported, underscoring the urgent need for intervention. Reference [71] reported a higher level of Cr in drinking water in Bangladesh, indicating serious negative health implications. Another study narrated that 17.24% of all water samples had Cr levels higher than the acceptable guidelines on groundwater in China’s Guanzhong Basin [72]. Similarly, Cabral Pinto et al. [73] stated that water, vegetables, and soil in Portugal were heavily contaminated by Pb, Cd, Zn, and Cr, indicating considerably higher levels than the acceptable range.

There has been a high use of chromium-containing fertilizers and insecticides in GB, which resulted in chromium accumulation in soil and water. In agricultural areas treated with these herbicides, drainage and runoff may contaminate nearby water supplies. Vegetables and crops absorb a considerable amount of toxic metals through their root hairs, so these metals accumulate enormously in them. In the study region, livestock manure for the fertilization of fields and crops that have been supplemented with chromium can also result in an excess of chromium in drinking water.

### 3.6. Geo-Accumulation Index of Soil

**Figure 3:**
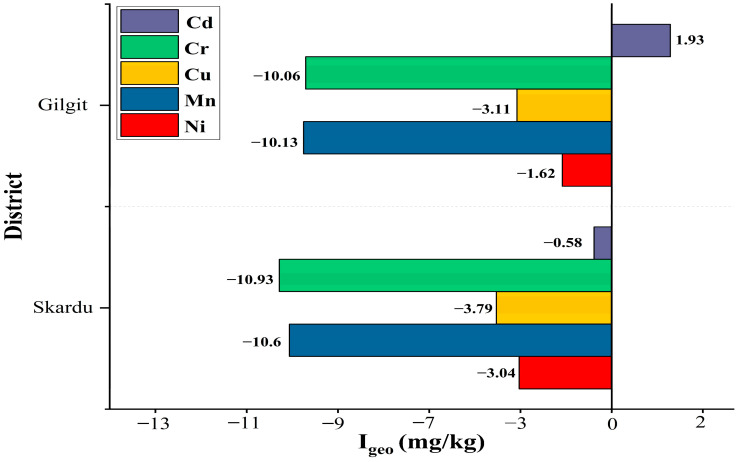
Geo-accumulation index of soil; a positive value was observed for Cd (1.93 mg/kg) from Gilgit, while all other elements show negative values for soil contamination.

The soil’s Geo-accumulation index for specific heavy metals in both districts revealed that the soil contamination status in Gilgit is satisfactory except for Cd (1.93 mg/kg) (Figure 3). Soil samples of the Skardu region showed noticeable non-contamination for all the heavy metals. It was found that the levels of Cd (−0.58 mg/kg) were slightly higher compared to other toxic metals. Table S2 presents the categorization of soils according to the level of pollution.

Fatna et al. [74] stated that Cd was the major toxic metal responsible for heavy contamination of the Geo-accumulation index and the source of contamination by multivariate factor analysis in Indonesia. The current results were also supported by the study conducted by Liu [75], who found higher levels in the surrounding soils of an electric factory in Jiaxing, China. Ara et al. [76], in a case study of leafy, fruit, and root vegetables, observed a significant level of Cd pollution in the following trend: Cd > Ar > Zn > Pb in the coastal region of Morocco. Another study revealed that Cd and As exceeded the background value of the soil at a rate that was 100% higher than expected in the soil of an electroplating factory in Jiaxing, China [77].

The higher level of Cd in the soil may be due to several manufacturing processes responsible for the elevated levels of Cd, including those associated with batteries, metal plates, pigments, and polymers. Additionally, these activities contribute to the release of Cd into the atmosphere on a regular basis. There is a high likelihood of metal contamination in the soil in areas located near factories or other industrial areas. In agricultural soils, Cd may accumulate as a result of the deliberate application of phosphate fertilizers. Phosphate fertilizers may contain cadmium as an impurity.

Long-term application of these fertilizers may result in a significant increase in the Cd content in Gilgit soil. Drinking water, vegetables, and soil can become contaminated with heavy metals, which can negatively impact ecosystems. Heavy metal accumulation decreases soil fertility by changing the organic matter content and pH and limiting plant growth. Heavy metal accumulation disturbs microbial communities and can have a toxic effect on beneficial microorganisms in soil that are involved in the nitrogen cycle, organic matter decomposition, and nitrogen fixation [78,79]. Metals taken up by plants can interfere with plant growth and disrupt enzyme activity and interfere with photosynthesis. Herbivores and the entire ecosystem are affected by these contaminated plants as they enter the food chain. The bioaccumulation of heavy metals in fish and aquatic organisms has a significant impact on their growth, reproduction, and survival [80,81].

In addition to having indirect or direct socioeconomic implications, heavy metal contamination may have a significant impact on local communities and industries. A substantial long-term exposure to heavy metals via drinking water, crops, and soil can lead to serious health concerns, such as neurological effects, organ failure, or cancer, particularly in vulnerable groups such as children [82]. Heavy metal accumulation not only affects soil productivity but also affects the food supply chain and the livelihood of farmers [83]. Agricultural exports from these regions are affected by the toxicity of heavy metals, which pose a health risk to livestock and crops, affecting food safety. Water, crops, and soil that contain heavy metals are major economic burdens that require extensive remediation techniques. Regular monitoring and maintenance of the water supply, local crops, and soil evaluation is required to safeguard public health and protect ecosystems [84,85].

### 3.7. Correlation Coefficient Matrix and PCA of Heavy Metals

**Figure 4:**
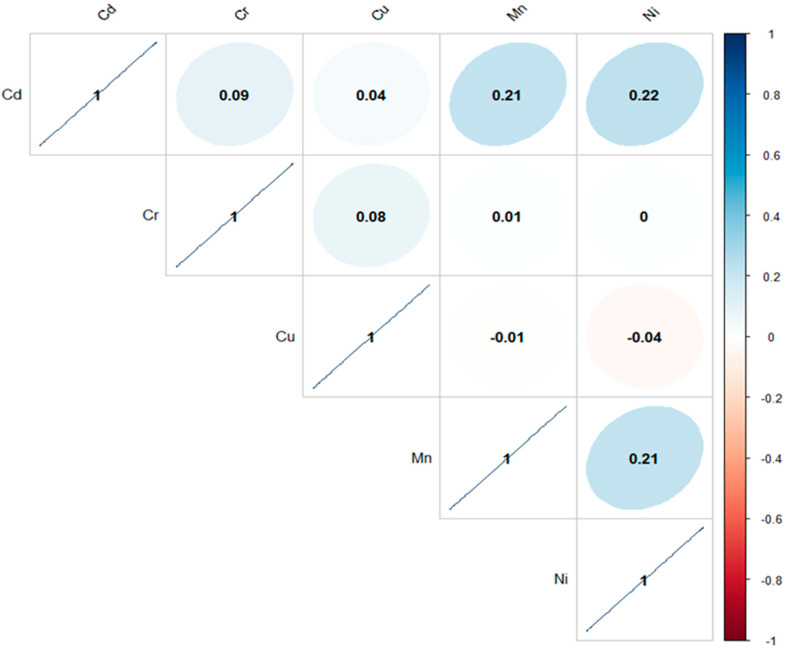
Correlation coefficient matrix of toxic metals; a dark blue color shows strong positive relations among metals, and a dark red color shows significant (*p* < 0.05) negative relations, while a white color shows neutral relations.

A correlation coefficient indicates the degree of linear relationship between any two variables, ranging from −1 (complete inverse association) through 0 (neutral association) to +1 (positive association), respectively. In the current study, the correlation coefficient was determined by applying the corr package to the data in R Studio (Figure 4). It was found that a strong and positive significant correlation existed between Mn/Cd, Ni/Cd, and Mn/Ni, whereas a robust and negative correlation subsists between Cu/Ni and Mn/Cu, and a neutral relationship was found in Ni/Cr. The correlation coefficient matrix is presented in Table S4. The negative correlation might suggest that the metals came from separate origins and sources or have dissimilarities; meanwhile, strong and substantial positive correlations suggested comparable genetic origins, chemical affinity, and/or common background values in the samples [76].

**Figure 5:**
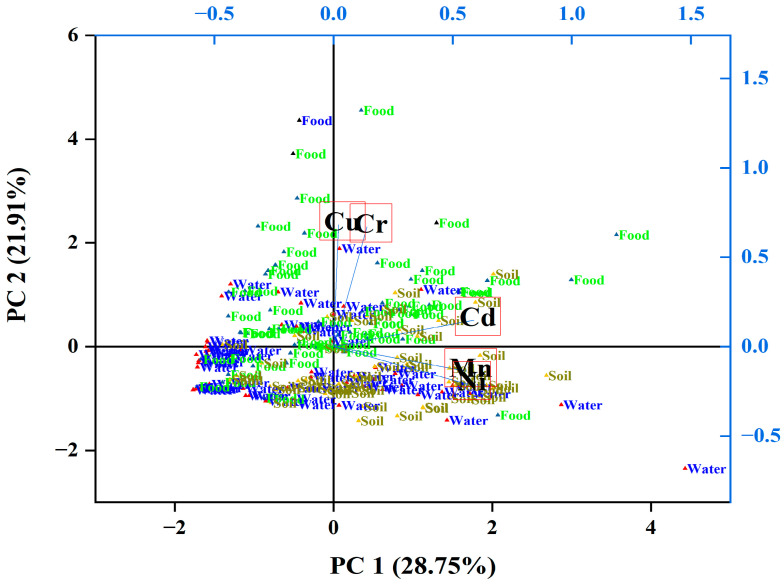
Principal component analysis of toxic metals; major sources of heavy metals were farming activities. Mn/Ni and Cu/Cr have similar origins, while Cd shows no positive relation with other elements.

Principle component analysis was performed to determine the hypothetical sources of heavy metals (natural or human) in the drinking water, vegetables, and soil by following the standard protocol [86,87] shown in Figure 5. The PCA was obtained, and its variance was demonstrated by 28.75% and 21.91% for the current study. Generally, PCA exposed two major components of five toxic metals examined in drinking water, vegetables, and soil. PCA1 was highly coevolved with Cd, Cu, Cr, Ni, and Mn, while PCA 2 was substantially coevolved with Mn and Ni (Table S5). The sources of PC1 and PC2, particularly manufacturing pollutants and the farming operations across the research area, might be viewed as mixed sources of human input. Burning fuel and coal generates vehicle emissions and environmental reserves, and it is thought that, in mountainous areas, metals have been gathered in these samples. According to PCA, the strength of identical toxic metals in drinking water, vegetables, and soil varies, because plants absorb and release these elements into the atmosphere.

In Gilgit-Baltistan, heavy metals are accumulated primarily due to natural geological features; other possible sources include mining operations, human activities, and agricultural practices that lead to soil and water contamination. The current study is supported by previous studies conducted in a similar geological region. According to a study conducted by Fatima et al. (2022), anthropogenic practices are significantly degrading the water quality in Bashu Valley in the District of Skardu [88]. Haq et al. (2023) concluded in another study conducted in the Ghizer River Basin that poor water quality may be associated with local activities of mountain communities, such as the discharge of household wastewater and the geology of the bedrock area [89]. Heavy metal accumulation has been significantly linked to high population density, extensive agricultural activities, and soil erosion in Shigar Valley, Skardu. This poses a potential health risk to residents of this area [90]. The geological attributes of the GB have led to excessive levels of heavy metals in the drinking water sources, as stated by Hussain et al. (2022) [46]. A subsequent study by Saira et al. (2019) suggested that drinking water contamination from nonpoint pollution sources; particularly, extensive fertilizer use should be urgently controlled and monitored for the protection of public health [91].

### 3.8. Health Risk Assessment

Based on the high cancer incidence rate in GB, Pakistan [17], it is imperative that toxic metals be assessed for their human health risk. As a result of their poisonous and cancer-causing properties, these heavy metals pose a serious health risk at high concentrations. In general, it is known that the carcinogens Cd and Cr are responsible for lung, urinary tract, and prostate cancers [92]. Exposure to Ni has been associated with lung and nasal malignancies. Excessive levels of Cu and Mn can also cause oxidative stress and damage to cells, which may ultimately result in cancer [93,94]. Possible contributors of these toxic metals in GB include mining operations, anthropogenic activities, and soil and water contamination due to agricultural practices.

A cancer registry is an online platform that compiles patient data and summarizes it into a clear summary of the patient’s health history, diagnosis, treatment plan, and present condition. The Directorate of Health Department Gilgit-Baltistan provided us with the cancer registry. This is a record of the instances that the government was monitoring; a surprisingly high number of cases were treated in private medical facilities. There were reports of about 60, 18, 26, 8, 22, 3, and 4 cases in Gilgit, Skardu, Ghizer, Hunza, Daimer, Nagar, and Astore. Thus, by examining non-carcinogenic and carcinogenic risk assessments, our goal was to identify the primary cancer-causing risk factors in the target area.

#### 3.8.1. Non-Carcinogenic Risk Assessment

**Figure 6:**
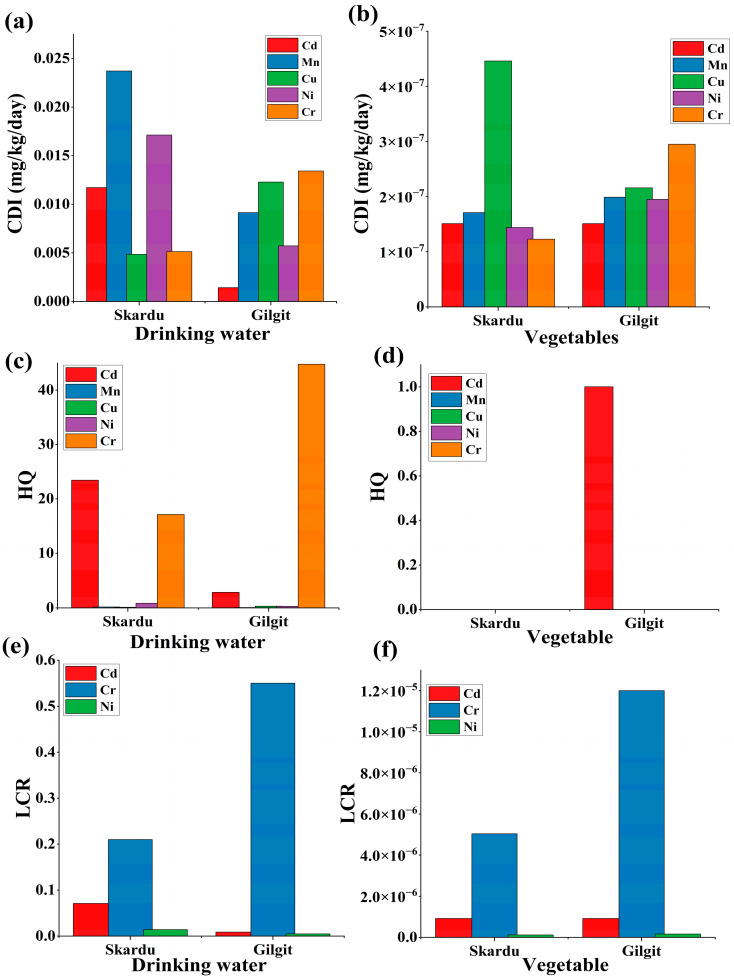
Risk assessment of heavy metals in various samples. (**a**) Chronic daily intake of toxic metals from drinking water. (**b**) Chronic daily intake of toxic metals from vegetables. (**c**) Hazard quotient level exceeded for Cr and Cd in water samples in Gilgit and Skardu. (**d**) Hazard quotient level of Cd in the vegetable sample from Gilgit. (**e**) LCR was higher for Cr, Cd, and Ni in drinking water from both regions, and (**f**) LCR from vegetables of both districts was within the allowable limit (>1 × 10^−4^).

The non-carcinogenic risk of selected heavy metals assessed by the target hazard quotient is presented in Figure 6. The results obtained indicated that higher levels of Mn 0.02372 mg/L/day, Ni 0.01714 mg/L/day, and Cd 0.01172 mg/L/day were recorded in drinking water samples from the Skardu region; meanwhile, Cr 0.01343 mg/L/day and Cu at 0.01229 mg/L/day had higher levels from Gilgit drinking water samples. These results indicated that the drinking water was heavily contaminated by Cd, Cr, and Ni, posing considerable adverse health risks to the adults. The vegetable samples showed low values for CDI compared to drinking water. The CDI order for the vegetable sample was observed as Cr > Cu > Mn > Ni > Cd; Cu > Mn > Cd > Ni > Cr mg/kg/day, respectively, for Gilgit and Skardu, as shown in Figure 6a,b.

The values for the hazard quotient showed a significantly higher level (HQ > 1) of Cd and Cr from both districts, indicating a noticeable non-carcinogenic risk in the population. The required oral reference dose (RfD) values for HQ was shown in Table 2. The observed HQ in the drinking water of Skardu was noted as Cd 23.44 and Cr 17.13; subsequently, the HQ for drinking water of Gilgit showed Cd 2.86 and Cr 44.77, respectively. The cumulative hazard index (HI) in drinking water samples was recorded as 41.7 and 48.27 for Skardu and Gilgit Districts, indicating severe public health concern. Compared to studies conducted in other mountainous regions, the HI levels observed in Skardu and Gilgit are among the highest reported, underscoring the urgent need for intervention. It was recorded that the HQ values for vegetables were under the safe limit (HQ < 1), except for Cd from vegetables in Gilgit District (Figure 6c,d). 

The non-carcinogenic risk assessment of heavy metals via three main exposure routes, such as ingestion, inhalation, and dermal, of soil particles was calculated. For each of the exposure routes, inhalation was the most frequent means of vulnerability. The pattern observed in the study was as follows: HQ_ing_ for soil samples showed higher values (HQ > 1) as compared to HQ_derm_ and HQ_inh_, and the order was recorded as HQ_ing_ > HQ_derm_ > HQ_inh_ for Cr, Mn, Cd, and Ni (Table 3 and Table 4). Alsafran [95] reported similar results on the health risk index in drinking water for Cr, Ni, and As, which exceeded the acceptable limit. Similarly, another study also noted HQ > 1 in soil from mining areas in South Africa [52]. Another study on wells and springs found higher values of health risks (HQ > 1) for Cr, Cd, Fe, and Mn, indicating that long-term, chronic consumption of these water sources poses a risk to an individual’s health [97]. The higher HQ values in drinking water of Gilgit and Skardu Districts may be due to the extensive application of fertilizer in field and crops, which ultimately run off into near water channels and streams. Thus, filtration plants for drinking water in areas with high levels of heavy metals should be established. The evaluation of potentially toxic heavy metals via three major exposure pathways is given below.

#### 3.8.2. Carcinogenic Risk Assessment

The lifetime cancer risk due to exposure to heavy metals (Cd, Mn, Cu, Ni, and Cr) through the consumption of vegetables and intake of drinking water was evaluated by computing the CDI values and cancer slope factor (CSF), as shown in Figure 6e,f. Oral reference dose and cancer slope factor of selected metals in water and vegetables are presented in Table 2. It was noticed that carcinogenic risk was found to be higher in drinking water for Cr and Cd, followed by Ni. The LCR for Cd was found to be 8.72 in 1000, 71.492 in 1000, Cr 550.63 in 1000, and 210.74 in 1000, indicating a significant cancer risk in drinking water samples from both districts. On the other hand, the LCR values for vegetable samples were found to be low risk < 1 × 10^−4^ for Cd, Cr, and Ni, respectively, from Gilgit and Skardu Districts.

In the case of soil as presented in Table 3 and Table 4, the LCR_ing_ and LCR_der_ were recorded as unacceptable < 1 × 10^−4^ to 1 × 10^−5^, and LCR_inha_ for Cd, Cr, and Ni was noted at 1.071 × 10^−5^, 2.4864 × 10^−7^, 1.3776 × 10^−6^ and 1.864 × 10^−7^, 2.158 × 10^−7^, 4.146 × 10^−6^, respectively, from Gilgit and Skardu. The toxicity response as oral reference dose and cancer slope factor of selected metals in the soil sample is presented in Table S3. The findings demonstrated that cancer risk from the soil via ingestion was greater than the safe limit between 10^−6^ < LCR < 10^−4^ as compared to the dermal and inhalation routes [98]. It was found that Ni exposure via LCR_ing_ was higher from the Gilgit region, and LCR_ing_ showed a significantly higher lifetime cancer risk to Cd as compared to Ni and Cr from the Skardu region. Kazemi et al. [99] reported that the LCR caused by Cd and Cr in Tehran drinking water outstripped the safe level, which might be a potential reason for carcinogenic risk in the community. Comparable findings were also narrated by Li et al. [100], who concluded that a risk of developing cancer by Cr and Cd was observed in terminal tap water in South China; however, As and Pb were within the permissible limits. Taghavi et al. [101] found Cr and Ni levels of 84.4 and 100% reported in HQ < 1 and CR > 1 within the range of 13 × 10^−2^ and 1.2 × 10^−3^, respectively.

Qasemi et al. [102] revealed that adults may be at risk of cancer from drinking water, indicating a carcinogenic risk greater than 10^−4^ in 42% and 16% of the rural regions of Bajestan and Gonabad Iran. Kumar et al. [49] highlighted the elevated carcinogenic risk posed by Cr, Cd, Ni, and As due to the intake of vegetables and grain in the populous city of Lucknow, India.

The possible reasons for the surplus values of LCR for Cd, Cr, and Ni might be due to the mining activities and mineral-rich rocks and mountains in Gilgit-Baltistan. Hazardous metals can be released into neighboring water bodies during mining operations due to runoff and poor waste management. Likewise, the excess accumulation of heavy metals in water and soil was due to the application of fertilizer, as well as pesticides, used in agricultural practices. These substances frequently include contaminants like Cr, Cd, and Ni that can build up in water bodies due to runoff from fields. When metal-containing items (including paints, detergents, and plumbing supplies) are frequently used in the home, the discharge of toxic metals in wastewater can rise and, if left untreated, can eventually find its way into natural water bodies. 

The results of the study indicated elevated levels of Cd, Mn, Cr, and Ni in drinking water and vegetables, exceeding permissible limits. Considering the fact that the population of Gilgit-Baltistan relies on natural water sources, local crops, and mining for their livelihoods, excessive heavy metal accumulation has been a significant issue. The mountain community is totally dependent on domestic farming and natural water resources due to harsh climatic conditions and the lack of infrastructure for the treatment of water. There is a significant association between chronic exposure to these heavy metals and severe health outcomes such as carcinogenic and non-carcinogenic health risks. The present study found significant health risks associated with Cr and Cd in drinking water, including non-carcinogenic (HQ > 1) and excess lifetime cancer risk > 1 × 10^−4^.

In agriculture, elevated levels of heavy metals in soil result in easy uptake by plants, which not only deteriorates the plant quality but also presents bioaccumulation risks to food chains. Furthermore, during flood season, farmers used muddy water for irrigation and agricultural practices, which contained large amounts of suspended solids with elevated levels of heavy metals. After they settle down, these heavy metals cause soil contamination. It was observed that contamination may occur due to mineral-rich sediments from glacier meltwater, extensive application of fertilizer and pesticides to crops, and excess use of livestock manure without following good agricultural practices. Due to these multiple risk factors, agrarian sustainability in the region has been diminished and highly vulnerable.

## 4. Study Limitation

One limitation of the current study is the potential for sampling bias, as the random sampling method may not fully capture the variability in contamination levels across the districts. The Monte Carlo simulation, although robust, is based on several assumptions that introduce uncertainty into the risk estimates. Longitudinal studies to evaluate chronic health effects and epidemiological surveys should be promoted to introduce a baseline for health conditions associated with heavy metals. Furthermore, future studies should aim to include a larger and more diverse sample set and explore the use of alternative probabilistic models.

## 5. Future Research and Direction

As shown in Table 5, only three investigations were conducted on the buildup of heavy metals in the previous years in Gilgit-Baltistan. Despite noticing an excessive amount of hazardous heavy metals in the source and a serious risk to human health, two studies were conducted on drinking water and one on soil and vegetables. While there is currently very little data available in the literature, this region is the most vulnerable in terms of access to safe food and quality healthcare. Therefore, future research may be focused on particular hazards and their relationship with health problems.

## 6. Conclusions

The current study was carried out to evaluate the cancer and non-cancer risks associated with potentially toxic metals in three exposure pathways, i.e., drinking water, vegetables, and soil from the Northern Areas of Pakistan. The findings revealed that the mean concentration of selected heavy metals (Cd, Mn, Cu, Ni, and Cr) in drinking water and vegetable samples exceeded the allowable limit set by the USEPA and WHO. The order for the CDI (mg/kg/day) in drinking water was Mn > Ni > Cd > Cr > Cu, recorded in Gilgit District and Mn > Ni > Cr > Cu > Cd in Skardu. In contrast, both districts have relatively low values for the CDI (mg/kg/day) in the vegetable samples. The order for the average concentration of heavy metals in the soil samples was as follows: Mn > Cd > Ni > Cu > Cr mg/kg/day, respectively. The hazard quotient values of Cd and Cr were more than 1 for both water samples, which indicated considerable adverse non-carcinogenic health outcomes in the community. In contrast, all vegetable samples showed a HQ less than 1. The cumulative HI for drinking water from Gilgit and Skardu was recorded as 48.27 and 41.7, respectively, indicating significant non-carcinogenic health risks.

It was found that HI > 1 is due to oral exposure to soil rather than dermal and inhalation exposure from both districts. The Geo-accumulation index indicates the pollution status of the soil. The order for Igeo observed was Cd > Ni > Cu > Mn > Cr, respectively. The Geo-accumulation index of soil indicated Cd (1.936 mg/kg) as the source of soil contamination in Gilgit. The finding revealed that the cancer risk from vegetable samples was lower as compared to drinking water. Based on the current outcomes, Cr and Cd were found to be more likely to cause health risks. LCR was above the safe limit due to inhalation exposure pathways from both regions as compared to oral and dermal ways, indicating noteworthy cancer risk to the population. Therefore, the current study suggests that effective monitoring measures be urgently taken to eliminate toxic metals concentrations, especially Cd, Ni, and Cr, from drinking water sources in both districts to safeguard the public health.

## Figures and Tables

**Table 1:** Analytical attributes of heavy metal analysis from various samples.

	Heavy Metals	LoD (mg/kg)	LoQ (mg/kg)	Recovery (%)
Vegetable	Cd	0.0032	0.014	96.65
	Cr	0.006	0.022	97
	Cu	0.004	0.017	102
	Mn	0.02	0.19	98
	Ni	0.05	0.86	95
Drinking water	Cd	0.0007	0.003	92
	Cr	0.002	0.0074	106
	Cu	0.011	0.063	93.21
	Mn	0.004	0.051	104
	Ni	0.0089	0.021	105
Soil	Cd	0.026	0.078	94
	Cr	0.101	0.432	99
	Cu	0.450	1.09	98.3
	Mn	1.17	3.04	99
	Ni	0.21	0.86	97

**Table 2:** Oral reference dose and cancer slope factor of selected metals in water and vegetables [95,96].

Toxicity Response	Cd	Cr	Ni	Mn	Cu
Oral reference dose	0.0005	0.0003	0.02	0.14	0.04
Cancer slope factor	6.1	41	0.84	-	-

-: not available.

**Table 3:** Risk evaluation of heavy metal in soil (Gilgit) via various exposure pathways.

Risk Assessment	Exposure Pathway	Cd	Cr	Cu	Mn	Ni
CDI mg/kg/day	Ingestion	1.18	0.411	0.547	1.135	1.136
	Dermal	4.84 × 10^−5^	1.36 × 10^−5^	1.81 × 10^−5^	3.80 × 10^−5^	3.75 × 10^−5^
	Inhalation	1.70 × 10^−7^	5.92 × 10^−8^	7.90 × 10^−8^	1.66 × 10^−7^	1.64 × 10^−7^
HQ	Ingestion	1180	137	13.675	56.75	56.8
	Dermal	0.0484	4.53 × 10^−5^	4.525 × 10^−4^	0.0019	6.94 × 10^−4^
	Inhalation	0.00017	1.97 × 10^−5^	1.975 × 10^−6^	8.3 × 10^−6^	7.961 × 10^−6^
LCR	Ingestion	0.59	0.2059	-	-	1.9312
	Dermal	2952 × 10^−4^	2.72 × 10^−5^	-	-	7.5 × 10^−5^
	Inhalation	1.071 × 10^−5^	2.4864 × 10^−7^	-	-	1.3776 × 10^−6^

CDI: chronic daily intake, HQ: hazard quotient, LCR: lifetime cancer risk, and -: not available.

**Table 4:** Risk evaluation of heavy metal in soil (Skardu) via various exposure pathways.

Risk Assessment	Exposure Pathway	Cd	Cr	Cu	Mn	Ni
CDI mg/kg/day	Ingestion	0.205	0.356	0.342	1.328	0.424
	Dermal	2.32 × 10^−5^	2.54 × 10^−5^	2.47 × 10^−5^	6.12 × 10^−5^	2.55 × 10^−5^
	Inhalation	2.96 × 10^−8^	5.14 × 10^−8^	4.93 × 10^−8^	1.93 × 10^−7^	4.94 × 10^−7^
HQ	Ingestion	205	118.67	8.55	66.4	21.2
	Dermal	0.0232	8.47 × 10^−3^	6.175 × 10^−4^	0.00306	4.722 × 10^−3^
	Inhalation	2.96 × 10^−5^	1.71 × 10^−5^	4.937 × 10^−5^	9.65 × 10^−6^	7.961 × 10^−6^
LCR	Ingestion	1.1025	0.1783	-	-	0.7208
	Dermal	11.415 × 10^−4^	5.08 × 10^−5^	-	-	5.1 × 10^−5^
	Inhalation	1.864 × 10^−7^	2.158 × 10^−7^	-	-	4.146 × 10^−6^

CDI: chronic daily intake, HQ: hazard quotient, LCR: lifetime cancer risk, and -: not available.

**Table 5:** Review of the existing studies on the accumulation of heavy metals in drinking water, soil, and vegetable samples in the Northern Areas of Pakistan.

Sample Type	Location	Technique	Risk Assessment Indices	Ni mg/kg	Ba mg/kg	Mn mg/kg	Cd mg/kg	Sb mg/kg	As mg/kg	Cr mg/kg	B mg/kg	Zn mg/kg	Fe mg/kg	Al mg/kg	Cu mg/kg	Hg mg/kg	Se mg/kg	Pb mg/kg	Reference
Drinking water	Gilgit	AAS	CDI, HQ, HI, CR	0.20	-	0.32	0.05	-	-	0.47	-	-	-	-	0.43	-	-	-	Current study
	Skardu			0.6		0.83	0.41			0.18					0.17				
Vegetables	Gilgit	AAS	CDI, HQ, HI, CR	0.58	-	0.57	0.44	-	-	0.86	-	-	-	-	0.63	-	-	-	Current study
	Skardu			0.42		0.50	0.44			0.36					1.3				
Soil	Gilgit	AAS	CDI, HQ, HI, CR	0.83	-	0.84	0.86	-	-	0.30	-	-	-	-	0.40	-	-	-	Current study
	Skardu			0.31		0.97	0.15			0.26					0.25				
Drinking water	Central Hunza	AAS	CDI, HQ	-	-	-	-	0.2700	-	0.2885	-	2.684	0.0580	-	0.5444	0.0046	-	0.1150	[91]
Drinking water	Skardu, Hunza Nagar, Astore, Gilgit, Ghizer, Diamer	ICP-OES GFAAS HGAAS	CDI, HQ	ND	0.017	ND	-	0.001	0.18	ND	0.032	0.01	-	ND	ND	0.009	ND	-	[31]
				ND	0.007	ND		ND	0.004	ND	0.003	ND		ND	ND	ND	ND		
				0.025	0.121	0.188		ND	0.004	0.033	0.528	0.176		16.653	0.015	0.00066	ND		
				ND	0.01	ND		0.0045	0.00074	ND	ND	ND		ND	ND	0.0025	ND		
				ND	0.004	0.0733		ND	0.000544	ND	ND	0.0144		ND	ND	0.0002	0.00022		
				ND	0.006	0.086		ND	0.0015	ND	ND	0.017		ND	ND	0.000074	ND		
				ND	0.0125	ND		ND	0.007	0.0155	ND	ND		ND	ND	ND	ND		
Soil	Kondodas Dainyor Nagril Jager-Baseen Naltar	-	DMI PTF HRI	52	-	-	-	-	-	1.0	-	590	-	-	147	-	-	35	[103]
				31						0.85		1193			99			36	
				36						0.3		172			55			43	
				57						0.75					72			29	
										2.3		210							
				24						74		460			71			138	
*M. sylvestris*	Gilgit area			10			0.24					247			20			20	
*B. Campestris*				04			0.62					271			17			17	
*S. Oleracea*				07			2.10					40			11			18	
*M. sylvestris*				20			0.67					78			28			44	
*C. Intybus*				12			0.81					240			48			42	
*T. Repens*				12			0.86					50			15			18	
*A. Viridis*				11			0.79					50			17			16	
*P. Oleracea*				10			0.94					96			09			16	
*B. Oleracea*				24			0.72					115			17			35	
*L. Sativa*				11			0.84					54			24			15	
*M. neglecta*				15			1.55					07			75			10	

ICP-OES: inductively coupled plasma-optical emission spectrometry, GFAAS: graphite furnace atomic absorption spectrometry, HGAAS: hydride generation atomic absorption spectrometry, DMI: daily intake of metal, PTF: plant transfer factor, and HRI: health risk index.

## Data Availability

Access to the current data will be available online (see Supplementary Material), while additional data will be provided upon request.

## Supplementary Materials

The following are available online at https://www.mdpi.com/article/10.3390/toxics13010005/s1, Table S1. The parameter used in CDI (mg/kg/day) for different exposure pathways in soil, Table S2. Taxonomy of soil, Table S3. Toxicity response as RfD and CSF in soil, Table S4: Correlation Coefficient Matrix of selected heavy metals, Table S5: Extracted Eigenvectors for PCA analysis.
