# Supplementary material for: Evaluating the Carcinogenic and Non-Carcinogenic Health Risks of Heavy Metals Contamination in Drinking Water, Vegetables, and Soil from Gilgit-Baltistan, Pakistan
Source: Toxics. 2024 Dec 25;13(1):5. doi: 10.3390/toxics13010005 (PMC11769479; doi:10.3390/toxics13010005)
Supplement: Supplementary file 1 [file toxics-13-00005-s001.zip › toxics-3360806-supplementary.pdf]

Evaluating the Carcinogenic and Non-Carcinogenic Health Risks of Heavy Metals Contamination in Drinking Water, Vegetables, and Soil from Gilgit-Baltistan, Pakistan

Nafeesa Khatoon [1, 2], Sartaj Ali [3], Azhar Hussain[3], Jia Huang [1], Zengli Yu [2*], Hongyan Liu [1*]

[1] Department of Medical Genetics, Henan Provincial People's Hospital, People's Hospital of Zhengzhou University, Zhengzhou 450003, China; nafeesaali.ft1354@gmail.com (N.K.); xxhj0712@126.com (J.H.)

[2] College of Public Health, Zhengzhou University, Zhengzhou 450001, China

[3] Department of Agriculture & Food Technology, Karakorum International University Gilgit, Gilgit 15100, Pakistan; sartaj@kiu.edu.pk (S.A.); azhar.hussain@kiu.edu.pk (A.H.)

*Correspondence: zly@zzu.edu.cn (Z.Y.); liuhongyanqhhy@126.com (H.L.)

Table S1. The parameter used in CDI (mg/kg/day) for different exposure pathways in soil.

Parameter	Terminology	Value	Unit	Reference
C	Level of metal	Observed value	mg/kg	
IR	Ingestion rate of soil	100	mg/kg	[47]
EF	Exposure frequency	350	days/yr	[48]
SA	Exposure surface of skin	5700	cm^2	[49]
SAF	Skin adherence factor	0.07	mg/cm^2	[47]
IRinh	Inhalation rate of soil	20	mg/cm^2	[46]
ABS	Dermal absorption factor	0.001	-	[50]
PEF	Particle emission factor	1.39×10^9	m^3/kg	[50, 51]

-; not available.

Table S2. Taxonomy of soil on the base of Geo accumulation index (I_{geo}).

Igeo	Pollution class
< 0	Uncontaminated
0 -1	Uncontaminated to moderate
1-2	Moderately contaminated
2-3	Moderate to heavily
3-4	heavily contaminated
4-5	Extremely contaminated
> 5	Very extreme level

Table S3. Toxicity response as oral reference dose and cancer slope factor of selected metals in soil sample [44, 46, 88].

Heavy metals	RFD_{ing} mg/kg/day	RFD_{derm} mg/kg/day	RFD_{inh} mg/kg/day	CSF_{ing} mg/kg/day	CSF_{derm} mg/kg/day	CSF_{inh} mg/kg/day
**Cd**	1×10^{-3}	2.5×10^{-5}	1×10^{-3}	5×10^{-1}	6.1	6.3
**Cr**	3×10^{-3}	3×10^{-3}	2.86×10^{-5}	5.01×10^{-1}	2	4.2

**Ni**	2×10^{-2}	5.4×10^{-3}	2.06×10^{-2}	1.7	2	8.4
**Mn**	2×10^{-2}	6×10^{-3}	1.43×10^{-5}	-	-	-
**Cu**	4×10^{-2}	1.2×10^{-2}	4.02×10^{-2}	-	-	-

RFD; Oral reference dose; CSF; cancer slope factor, -; not available.

Table S4: Correlation Coefficient Matrix of selected heavy metals

	**Cd**	**Cr**	**Cu**	**Mn**	**Ni**
**Cd**	1	0.09395	0.03931	**0.21291**	**0.22015**
**Cr**	0.09395	1	0.07531	0.00524	0.00305
**Cu**	0.03931	0.07531	1	-0.00549	-0.03791
**Ni**	**0.21291**	0.00524	-0.00549	1	**0.21046**
**Mn**	**0.22015**	0.00305	-0.03791	**0.21046**	1

Bold values indicate they have a strong positive relationship

Table S5: Extracted Eigenvectors for PCA analysis

Heavy metals	Coefficient of PCA1	Coefficient of PCA2
Cd	0.58903	0.14808
Cr	0.1408	0.67034
Cu	0.68219	0.68219
Mn	0.55995	-0.13731
Ni	0.565	-0.21092

Reference

44. US Environmental Protection Agency. *Framework for Metals Risk Assessment*; US Environmental Protection Agency: Washington, DC, USA, 2007.

46. Epa, U.S. *Exposure Factors Handbook*; EPA: Washington, DC, USA, 2011; Volume 20460, pp. 2–6.

47. Hussain, S.J.; Ali, S.; Hussain, J.; Ali, S.; Hussain, J.; Hussain, M.; Hussain, I. Contamination and Human Health Risk Assessment of Toxic Trace Elements in Drinking Water of Gilgit-Baltistan, Pakistan. *Pertanika J. Trop. Agric. Sci.* **2023**, *31*, 197–201.

48. Wang, Z.; Bao, J.; Wang, T.; Moryani, H.T.; Kang, W.; Zheng, J.; Zhan, C.; Xiao, W. Hazardous Heavy Metals Accumulation and Health Risk Assessment of Different Vegetable Species in Contaminated Soils from a Typical Mining City, Central China. *Int. J. Environ. Res. Public Health* **2021**, *18*, 2617.

49. Kazemi Moghaddam, V.; Latifi, P.; Darrudi, R.; Ghaleh Askari, S.; Mohammadi, A.A.; Marufi, N.; Javan, S. Heavy metal contaminated soil, water, and vegetables in northeastern Iran: Potential health risk factors. *J. Environ. Health Sci. Eng.* **2022**, *20*, 65–77. https://doi.org/10.1007/s40201-021-00756-0.

50. Khezerlou, A.; Dehghan, P.; Moosavy, M.-H.; Kochakkhani, H. Assessment of heavy metal contamination and the probabilistic risk via salad vegetable consumption in Tabriz, Iran. *Biol. Trace Elem. Res.* **2021**, *199*, 2779–2787.

51. Kumar, P.; Dipti; Kumar, S.; Singh, R.P. Severe contamination of carcinogenic heavy metals and metalloid in agroecosystems and their associated health risk assessment. *Environ. Pollut.* **2022**, *301*, 118953. https://doi.org/10.1016/j.envpol.2022.118953.

88. Franco-Uría, A.; López-Mateo, C.; Roca, E.; Fernández-Marcos, M.L. Source identification of heavy metals in pastureland by multivariate analysis in NW Spain. *J. Hazard. Mater.* **2009**, *165*, 1008–1015.
